# Effects of lactate concentration on T‐cell phenotype and mitochondrial respiration

**DOI:** 10.14814/phy2.70450

**Published:** 2025-07-24

**Authors:** Eunhan Cho, Guillaume Spielmann, Brian A. Irving

**Affiliations:** ^1^ School of Kinesiology Louisiana State University Baton Rouge Louisiana USA

**Keywords:** flow cytometry, high resolution respirometry, immunometabolism, L‐lactate, preconditioning, T‐cell bioenergetics

## Abstract

Lactate is a critical regulator of cellular processes and immune signaling, and we hypothesize that exercise‐induced elevations in lactate help activate immune cells in response to vigorous exercise. Despite its importance, the impact of lactate on T‐cell mitochondrial respiration remains poorly understood. This study examines the impact of exposure to physiologically relevant lactate concentrations (0.5 and 4.0 mM) on the mitochondrial respiration of resting T‐cells. Resting T‐cells were isolated from 12 healthy participants (mean ± SD, 26.8 ± 3.5 years) and cultured in a plasma‐like medium with either 0.5 mM (control) or 4 mM lactate for 1 h to mimic resting and vigorous exercise conditions. The composition of T‐cell subsets was characterized using flow cytometry, and mitochondrial respiration was measured using high‐resolution respirometry. Exposure to 4 mM lactate significantly increased mitochondrial oxygen flow (*I*_O2_, pmols∙s^−1^ million T‐cells^−1^) across all respiratory states compared to the control condition (0.5 mM) (all *p* < 0.01), suggesting an enhanced capacity for oxidative phosphorylation compared to the control. This study demonstrates that lactate preconditions T‐cells and leads to enhanced mitochondrial respiration, offering insights into immune cell metabolism under exercise‐like conditions, independent of exercise‐induced differential mobilization of immune cell subsets.

## Graphical abstract

Physiologically relevant lactate concentrations precondition T‐cells and lead to enhanced mitochondrial respiration.

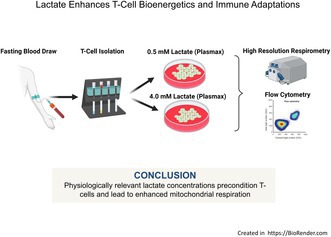

## 1 INTRODUCTION

Lactate, long considered a byproduct of anaerobic metabolism, is now recognized as the primary metabolic product of glycolysis, even under aerobic conditions, particularly in skeletal muscle (Brooks et al., 2023). Moreover, skeletal muscle releases lactate into the blood at rest, which is further accentuated during exercise, as demonstrated using stable‐isotope methodologies (Bergman et al., 1999; Messonnier et al., 2013; Miller et al., 2002). Once released into the bloodstream, lactate circulates to multiple tissues to serve as an essential metabolic intermediate (Brooks et al., 2023; Cai et al., 2023), and has more recently been recognized for its role as an exercise‐induced signaling molecule (e.g., exerkine) (Brooks et al., 2023). We hypothesize that exercise‐induced elevations in circulating lactate concentrations contribute to the activation of circulating immune cells, independently of immune compartment composition. For example, we have demonstrated that high‐intensity exercise evokes profound changes in the composition of circulating immune cells, including T‐cells (Spielmann et al., 2014), B‐cells (Turner et al., 2016), NK‐cells (Bigley et al., 2015; Cho et al., 2023), and Innate Lymphoid cells (Cho et al., 2021), which are associated with enhanced bioenergetic functions. Indeed, maximal exercise enhances routine respiration in peripheral blood mononuclear cells (PBMC) in collegiate swimmers (Stampley et al., 2023), and exercise above the lactate threshold increases the oxidative capacity (OXPHOS) of NK‐cells in healthy young adults (Cho et al., 2023). However, the studies above did not test the effect of elevated blood lactate concentrations on the bioenergetic functions of circulating immune cells, independently of cellular subset changes. Thus, the purpose of the present study was to use an *in vitro* experiment to examine the effect of a 1 hour exposure to low (0.5 mM) versus high (4.0 mM) physiologically relevant lactate concentrations on T‐cell phenotypes and bioenergetics, mimicking resting, and vigorous exercise conditions.

## 2 MATERIALS AND METHODS

### 2.1 Participants

The study included 12 young, healthy participants (26.8 ± 3.5 years). The cohort consisted of 5 males (42%) and 7 females (58%), with a racial distribution of 6 non‐Hispanic White participants (50%) and 6 non‐White participants (50%). Of which 3 were of Hispanic/Latino origin, 2 where of Asian origin and 1 was of African origin. Their physical activity level was moderate, as indicated by a Physical Activity Rating (PA‐R) of 5.6 ± 1.9 (Table 1). Individuals with metabolic, inflammatory, or cardiovascular diseases were excluded from this study.

### 2.2 Experimental design and procedures

The experimental design involved a single study visit. Prior to this visit, participants were instructed to refrain from vigorous exercise and fast for 12 h, consuming only water. Upon arrival, participants completed the informed consent process and a series of questionnaires, including the Physical Activity Readiness Questionnaire (PAR‐Q), a self‐reported medical history form, and a health history questionnaire. Additionally, height and weight were measured using a stadiometer and a calibrated scale. Finally, a fasted blood sample was collected in two 10‐mL EDTA vacutainers (BD, Franklin Lakes, NJ, USA).

### 2.3 Outcome assessment

#### 2.3.1 PBMC and T‐cell isolation

Complete blood counts were determined using an automated hematology analyzer in duplicate (Sysmex XN‐330, Sysmex Co., Kobe, Japan). Immediately after complete blood counting, peripheral blood mononuclear cells (PBMC) were isolated by density gradient centrifugation as previously described (Stem Cell Technologies, Vancouver, Canada, Cat#18060) (Cho et al., 2021). After isolation, PBMCs were counted using an automated cell counter (Countess 3 FL; ThermoFisher Scientific Inc.). Then, according to the manufacturer's instructions, T‐cells were isolated by negative selection using magnetic‐activated cell sorting (MACS) separation beads (Miltenyi Biotec, Auburn, CA, Cat#130‐096‐535). Negatively sorted T‐cells were washed with RPMI at 400 g for 10 min and then counted.

#### 2.3.2 Cell culture incubation

The isolated T‐cells were cultured for 1 h in plasma‐like media (Plasmax™, XimBio, London, UK, Cat#156371) containing either 0.5 or 4 mM lactate in a 37°C humidified incubator with 5% CO_2_. Plasmax™ is a cell culture medium designed to mimic human plasma by containing physiological concentrations of glucose, lactate, amino acids, micronutrients, inorganic salts, and other trace elements. To achieve the 4 mM lactate concentration, 3.5 mM sodium L‐lactate (Sigma: L7022) was added to the 0.5 mM lactate in Plasmax™, while 3.5 mM sodium chloride (Sigma: S7653) was added to the 0.5 mM lactate in Plasmax™ stock as a counter‐ion control (Lund et al., 2023). The osmolarity of the 0.5 and 4 mM lactate media was then adjusted to 290 mOsm/kg by adding purified water (Cytiva, HyClone). The final osmolarity was confirmed using an osmometer (Wescor, ELITechGroup). The pHs of the Plasmax Media with 0.5 and 4.0 mM L‐lactate were 7.45 and 7.65, respectively, after 1 h of incubation in 5% CO_2_.

#### 2.3.3 Antibody labeling and flow cytometry

Following a 1 h incubation, cells were stained using pre‐diluted monoclonal antibodies (mAbs) in a multi‐color direct immunofluorescence assay. The following fluorophore‐conjugated mAbs were utilized: anti‐CCR7 (Brilliant Violet 421, clone G043H7, BioLegend, Cat# 353208), anti‐CD45RA (cFluor v450, clone HI100, Cytek, Ref# R7‐20122), anti‐CD3 (BV570, clone UCHT1, BioLegend, Cat# 300436), anti‐CD4 (cFluor BYG584, clone SK3, Cytek, Ref# R7‐20042), anti‐CD28 (BV650, clone CD28.2, BioLegend, Cat# 302946), anti‐CD8 (cFluor B532, clone SK1, Cytek, Ref# R7‐20124), anti‐CD45 (cFluor R780, clone 2D1, Cytek, Ref# R7‐20134), anti‐CD27 (cFluor R840, clone QA17A18, Cytek, Ref# R7‐20082) and anti‐CD69 (PE‐Cy7, clone FN50, eBioscience, Ref# 25‐0699‐42). Ghost Dye Violet 510 (Cytek, Ref# 13‐0870‐T100) was used for viability staining.

Following antibody labeling, cellular phenotypes were assessed using a 3‐laser full‐spectrum flow cytometer (Aurora, Cytek Biosciences, Fremont, CA, USA). Data acquisition and spectral unmixing were performed using SpectroFlo® software (Cytek Biosciences), allowing for real‐time visualization. Subsequent data analyses were conducted using FCS Express (Version 7.0, De Novo Software, Pasadena, CA, USA).

#### 2.3.4 High‐resolution respirometry

Mitochondrial respiration was assessed using high‐resolution respirometry (Oroboros Instruments, Innsbruck, Austria). Initial room‐air calibration was performed in mitochondrial respiratory medium 5 (MiR05: 0.5 mM EGTA, 20 mM taurine, 3 mM MgCl_2_, 110 mM sucrose, 60 mM K‐lactobionate, 10 mM KH_2_PO_4_, 20 mM K‐HEPES, 1 g/L fatty acid‐free BSA, pH 7.1; MiR05 Kit, Oroboros: 60101‐01) at 37°C. Following calibration, 3–6 million T‐cells were added per 0.5 mL chamber. After stabilization, routine oxygen flow (*I*_O2_, pmols∙s^−1^∙million T‐cells^−1^) was measured in intact cells (CE). Cell permeabilization was achieved by titrating 30 μM α‐chaconine (Sigma: 80075). Next, we added palmitoyl‐L‐carnitine (Pal, 10 μM, Sigma: P1645) and malate (M, 1 mM, Sigma: M1000) to measure the *I*_O2_ in the LEAK state for fatty acid‐linked substrates (substrate state/pathway state: PalM_L_/F_L_). Next, we titrated ADP (2.5 mM, Sigma: A5285) with MgCl_2_∙6H_2_O (1.5 mM: Sigma: M2670) to measure the *I*_O2_ in the OXPHOS state for fatty acid‐linked substrates (PalM_P_/F_P_), followed by cytochrome c (cytc, 10 μM, Sigma C7752) to assess mitochondrial membrane integrity (PalM_P__cytc_/F_Pcytc_). Next, we titrated pyruvate (Pyr, 5 mM, Sigma: P2256) followed by glutamate (G, 10 mM, Sigma: G1626) to assess the additive effect of NADH‐linked respiration on the *I*_O2_ in the OXPHOS state (PalMPyr_P_/FN_P_ and PalMPyrG_P_/FN_P_). We then titrated succinate (S, 10 mM, Sigma: S2378) to measure the additive effect of S‐linked respiration on the *I*_O2_ in the OXPHOS state (PalMPyrGS_P_/FNS_P_). Subsequently, we titrated in rotenone (R, 0.5 μM, Sigma: R8875) to inhibit complex I, to measure S‐linked *I*_O2_ in the OXPHOS state (PalMPyrGSR_P_/S_P_). Finally, we titrated in antimycin A (2.5 μM, Sigma: A8674) to measure residual oxygen consumption (*R*_OX_), which was subtracted from all *I*_O2_ measurements. All experiments were performed at 37°C with oxygen concentrations maintained between ~190 and 50 μM O_2_. All *I*_O2_ measurements were corrected for daily air calibration, routine instrumental background calibrations and zero calibrations. Individual data points were the mean of duplicate measurements, except for one sample, which had one of its duplicate measurements excluded due to poor quality. In addition, the *I*_O2_ measured in the PalM_L_/F_L_ state for one participant was excluded due to unstable readings during this phase.

### 2.4 Statistical analysis

All statistical analyses were performed using JMPro 18 (SAS Inc., Cary, NC), and graphical representations were constructed using GraphPad Prism 10.00 (GraphPad Software). Linear Mixed Models were used to test the effects of different concentrations of Lactate (0.5 vs. 4 mM) on mitochondrial respiration and phenotypes. Data are presented as mean ± standard deviation (SD). Statistical significance was declared at *p* < 0.05.

## 3 RESULTS

### 3.1 The effects of lactate concentration on T‐cell phenotypic alteration

T‐cell phenotypes were quantified using flow cytometry and classified as Naïve, central memory (CM), effector memory (EM) and terminally differentiated effector memory cells re‐expressing CD45RA (TEMRA) subsets for both CD4^+^ and CD8^+^ T‐cells (Table 2). The proportion of naïve CD4^+^ T‐cells tended to decrease in 4 mM lactate concentration compared to 0.5 mM (0.5 mM: 48.48 ± 14.95% vs. 4 mM: 46.45 ± 15.31%; *p* = 0.077), while naïve CD8^+^ T‐cells were significantly lower in the high lactate concentration than in the low lactate condition (0.5 mM: 48.66 ± 14.11% vs. 4 mM: 46.63 ± 14.35%; *p* = 0.041). This reduction is likely associated with decreased expression of CCR7, a key marker in maintaining the naïve phenotype and migration capacity of T‐cells. However, all the other T‐cell phenotypes remained unchanged after a 1‐h incubation. Furthermore, a 1 h incubation did not induce T‐cell activation, as measured by the expression of the early activation marker CD69^+^ (Table 2).

**TABLE 1:** Demographic, physical, exercise characteristics of the participants.

Measurements	All (*n* = 12)
Age, years	26.8 ± 3.5
Male	5 (42%)
Female	7 (58%)
White	6 (50%)
Non‐white	6 (50%)
Height (cm)	174.8 ± 10.4
Weight (kg)	74.3 ± 16.5
BMI (kg/m^2^)	24.1 ± 3.4
Physical activity rating	5.6 ± 1.9

*Note*: Data are presented as mean ± SD or *n* (%).

**TABLE 2:** Cellular phenotypes were determined using flow‐cytometry in T‐cells that were incubated in plasma‐like media with either 0.5 or 4 mM L‐lactate for 1 h.

Phenotype (%)	0.5 mM	4 mM	*p*‐value
Naïve CD4^+^ (CCR7^+^/CD45RA^+^)	48.48 ± 14.95	46.45 ± 15.31	0.08
CM CD4^+^ (CCR7^+^/CD45RA^−^)	18.48 ± 5.26	18.13 ± 7.09	0.67
EM CD4^+^ (CCR7^−^/CD45RA^−^)	28.12 ± 11.66	29.66 ± 12.44	0.12
TEMRA CD4^+^ (CCR7^−^/CD45RA^+^)	4.90 ± 3.54	5.72 ± 3.75	0.19
Naïve CD8^+^ (CCR7^+^/CD45RA+)	48.66 ± 14.11	46.63 ± 14.35	0.04
CM CD8^+^ (CCR7^+^/CD45RA^−^)	2.03 ± 1.10	1.94 ± 1.00	0.57
EM CD8^+^ (CCR7^−^/CD45RA^−^)	18.17 ± 7.97	18.97 ± 8.36	0.18
TEMRA CD8^+^ (CCR7^−^/CD45RA^+^)	31.13 ± 13.15	32.45 ± 12.60	0.14
CD3^+^/CD4^+^/CD69^+^	0.66 ± 0.38	0.68 ± 0.37	0.63
CD3^+^/CD8^+^/CD69^+^	1.04 ± 0.64	1.00 ± 0.63	0.53

*Note*: *N* = 12, and data are presented as mean ± SD.

### 3.2 The effects of lactate concentration on T‐cell bioenergetics

**FIGURE 1:**
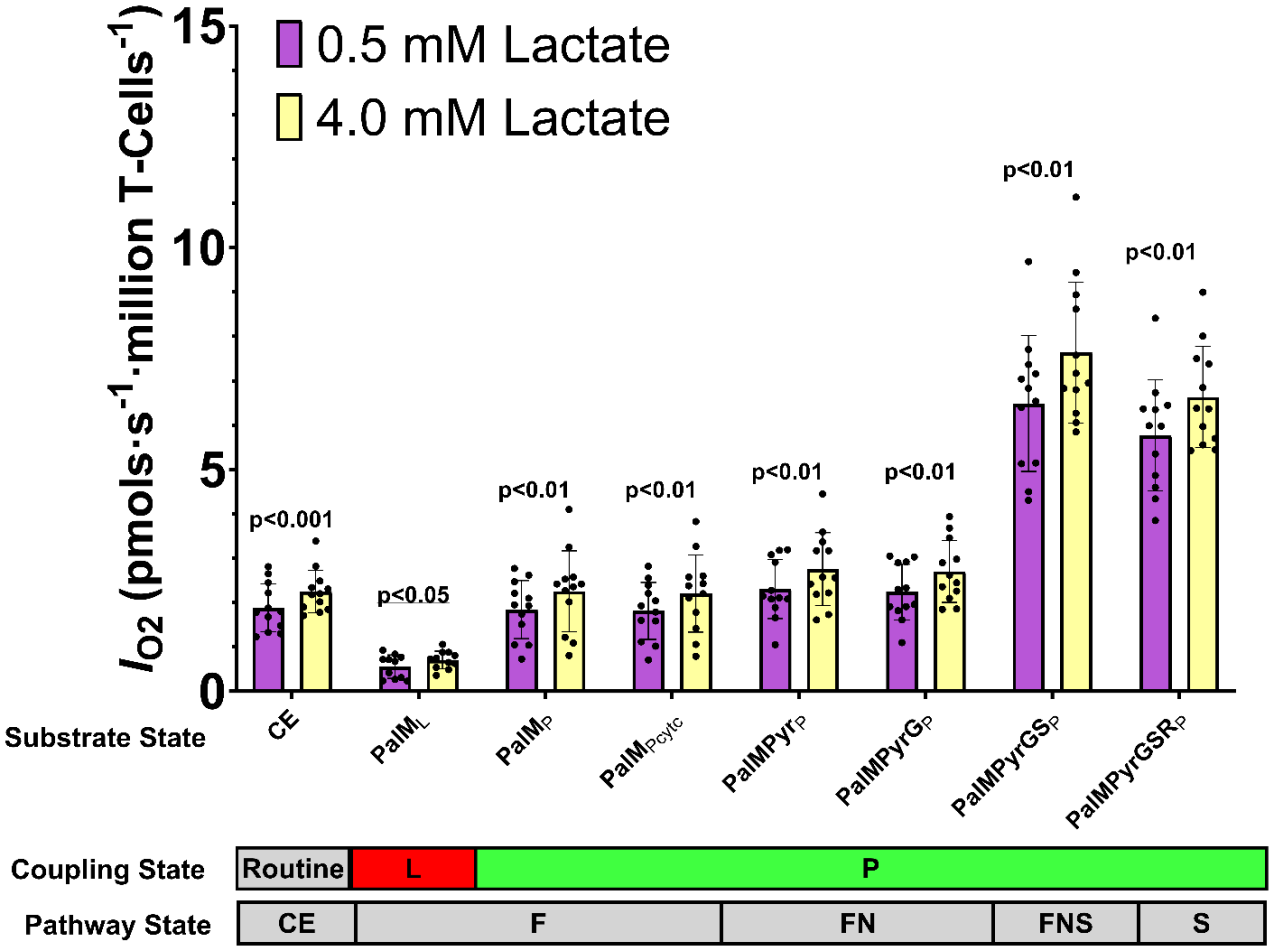
Cellular oxygen flow (*I*_O2_, pmols∙s^−1^∙million T‐cells^−1^) was measured in T‐cells that were incubated in plasma‐like media with either 0.5 mM or 4 mM L‐lactate for 1 h. The *I*_O2_ was measured in the intact cells (CE, routine), and chaconine‐permeabilized T‐cells under LEAK (L), and OXPHOS (P), coupling control states using endogenous (cells) and serial titrations of F‐Linked (PalM_L_: +10 µM palmitoyl‐L‐carnitine and 1.0 mM malate; PalM_P_: +2.5 mM ADP; PalM_Pcytc_: +10 µM cytochrome c), FN‐Linked (PalMPyr_P_: +5 mM pyruvate; PalMPyrG_P_: + 10 mM glutamate), FNS‐Linked (PalMPyrGS_P_: +10 mM succinate), S‐Linked (PalMPyrGSR_P_: +0.5 µM rotenone) substrates. LEAK was measured in the absence of ADP, and OXPHOS capacity was measured in the presence of 2.5 mM ADP. All data were background corrected for residual oxygen consumption measured in the presence of 2.5 µM antimycin A. *p*‐values are for comparison between 0.5 mM and 4 mM L‐lactate media from mixed model analyses. *N* = 12, data are presented as mean ± SD. The *I*_O2_ measured in PalM_L_/F_L_ state for one participant was excluded due to unstable readings during this phase.

**FIGURE 2:**
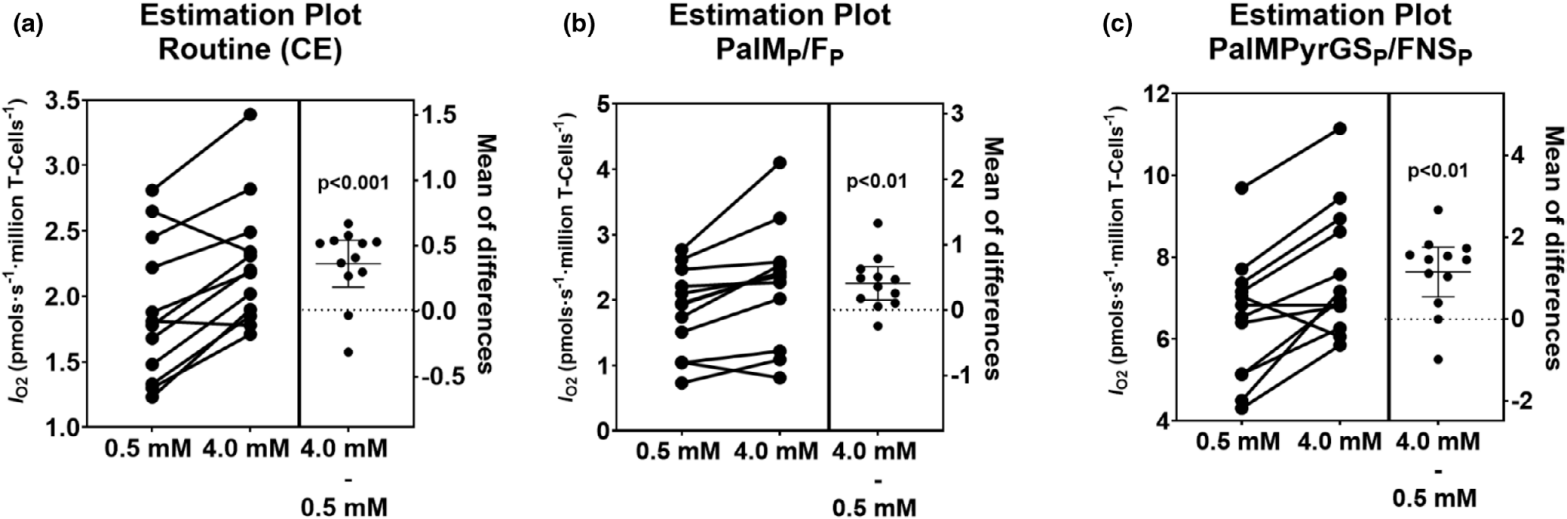
Cellular oxygen flow (*I*_O2_, pmols∙s^−1^∙million T‐cells^−1^) in T‐cells that were incubated in plasma‐like media with either 0.5 or 4 mM L‐lactate for 1 h, and the mean difference (95% confidence interval) between the two conditions. (a) Estimation plot of Routine (CE), (b) PalM_P_/*F*_P_, and (c) PalMPyrGS_P_/FNS_P_. *N* = 12 and *p*‐values are from mixed model analysis.

The effects of low (0.5 mM) and high (4 mM) lactate concentrations, mimicking resting and exercise‐like conditions, respectively, on T‐cell mitochondrial respiration were assessed using high‐resolution respirometry. Significant differences were observed across various substrate and pathway coupling control states (Figure 1, Table S1). Compared to the low lactate condition, routine *I*_O2_ in CE was 19.7% higher in the 4.0 mM lactate condition (Figure 1, Table S1, *p* = 0.0009). Compared to the low lactate condition, the *I*_O2_ in the LEAK state (PalM_L_/F_L_) was 48.4% higher in the 4.0 mM lactate condition (Figure 1, Table S1, *p* = 0.015). Notably, compared to the low lactate condition, the *I*_O2_ was 15.45%, 15.17%, 16.55%, 14.72%, and 12.79% higher in all OXPHOS states (PalM_P_/F_P_, PalMPyr_P_/FN_P_, PalMPyrG_P_/FN_P_, PalMPyrGS_P_/FNS_P_, PalMPyrGSR_P_/S_P_) in the 4 mM lactate conditions compared to the low lactate condition (Figure 1, Table S1, all *p* < 0.01). Figure 2 highlights the paired nature of our experimental protocol and demonstrates consistently higher *I*_O2_ in the 4.0 mM lactate condition compared to the low lactate condition for routine respiration (CE), fatty acid oxidation (PalM_P_/F_P_) and total OXPHOS (PalMPyrGS_P_/FNS_P_).

## 4 DISCUSSION

This study demonstrated that *in vitro* exposure of T‐cells to 4.0 mM lactate, a key metabolite released from skeletal muscle during exercise, can precondition T‐cells and lead to enhanced mitochondrial respiration relative to control (0.5 mM). By isolating the preconditioning effects of L‐lactate *in vitro*, we provide mechanistic insights into how exercise‐induced elevations in circulating L‐lactate (4 mM) may prime immune function independently of cellular subset redistribution. Relative to control, exposure to 4 mM lactate for 1 h in plasma‐like media significantly increased *I*_O2_ during all OXPHOS states (F_P_, FN_P_, FNS_P_ and S_P_), and thus increased respiration linked to ATP production in this study, which is consistent with the notion that lactate acts as both a mitochondrial substrate and a signaling molecule (Brooks et al., 2022; Brooks et al., 2023). Our findings corroborate a recent study showing that exposure to D‐lactate *in vitro* also elevates T‐cell bioenergetics (Cai et al., 2023). Notably, using the more physiologically relevant L‐lactate underscores its role in exercise‐mimicking conditions, stimulating oxidative phosphorylation (OXPHOS). Prior research has also shown that D‐lactate enhances the oxygen consumption rates associated with ATP synthesis while activating the electron transport system *in vitro*, which could elevate electron transport efficiency (Cai et al., 2023). While the underlying mechanism remains elusive, it could be related to the lactylation of electron transport proteins (Chen et al., 2025). In addition, lactate also likely indirectly regulates the electron transport system by changing the redox balance (Quinn 3rd et al., 2020). Consistent with the present results, a recent report also demonstrates that relative to basal (0.8 mM) L‐lactate concentrations, exposure to high (4 mM) L‐lactate concentrations also increased oxygen consumption rates of human regulatory T‐cells (Zhou et al., 2024). Since T‐cells express monocarboxylate transporters (MCT1 and MCT4), exogenous lactate is likely taken up by T‐cells and imported into their mitochondria to stimulate and/or regulate their respiration (D'aria et al., 2024; Barbieri et al., 2023; Brooks, 2009).

Our findings demonstrate that 4 mM lactate enhances mitochondrial respiration in resting T‐cells, indicating that this exercise‐responsive metabolite can modulate T‐cell bioenergetics. While the previous study in our lab showed that maximal exercise increased PBMC routine respiration in athletes (Stampley et al., 2023) and NK‐cell OXPHOS in response to exercise above the lactate threshold in inactive healthy women (Cho et al., 2023), these effects resulted from dynamic shifts in circulating cell populations, including a substantial mobilization of effector receptors and changes in NK‐cells and/or T‐cell subsets (Cho et al., 2023; Spielmann et al., 2014). Here, we demonstrated that exposure to 4.0 mM lactate for 1 h can precondition resting T‐cells and lead to enhanced respiratory function, further highlighting the role of L‐lactate as a key exercise‐derived metabolite that primes cellular energetics independently of the well‐established exercise‐induced changes in the circulating T‐cell pool composition. One potential mechanism by which L‐lactate can precondition the T‐cell pool is through the activation of hydroxycarboxylic acid receptor 1 (HCAR1), also known as GRP81, a key mediator of T‐cell immune activation (Feng et al., 2022; Su et al., 2024). Future mechanistic studies are needed to elucidate whether GPR81 regulates the effect of lactate preconditioning on T‐cell bioenergetics. In addition, one could postulate that elevations in L‐lactate could lead to a higher intracellular and intramitochondrial concentrations of L‐lactate and perhaps pyruvate, which could explain the higher routine (intact cell) respiration in T‐cells preconditioned with 4.0 mM L‐lactate compared to those preconditioned with 0.5 mM L‐lactate. However, it is unlikely that elevations in intracellular L‐lactate and/or pyruvate would lead to elevations in OXPHOS in permeabilized T‐cells. Specifically, elevations in intracellular L‐lactate and/or pyruvate, along with ADP, in the intact T‐cell would be washed out following permeabilization of the plasma membranes with chaconine. Finally, using a non‐metabolizable analogue to L‐lactate could potentially help distinguish lactate‐specific effects on cellular respiration from nonspecific osmotic or ionic changes. However, there does not appear to be a nontoxic, nonmetabolizable, natural L‐lactate analogue that could serve this purpose. As noted above, D‐Lactate is an unphysiological isoform of lactate, likely produced by some lactobacillus gut microbiota, which may contribute to lower bowel syndrome (Li et al., 2025). Moreover, elevations in D‐lactate have also been associated with the development of neurological disorders (Li et al., 2025). Alternatively, others have used ethyl lactate, but it is unnatural and physiologically inappropriate; it has been shown to inhibit the release of cytokines in human blood cells (Hollenbach et al., 2008).

Our results also revealed that high L‐lactate concentrations (4 mM) reduced CCR7 expression via receptor internalization or desensitization, relative to basal L‐lactate concentrations (0.5 mM). However, given that sodium lactate dampens CD4^+^ T‐cell motility *in vitro* by inhibiting CXCR3 signaling (Haas et al., 2015), it suggests that lactate may similarly trigger CCR7 internalization independently of its ligand, CCL19. The lack of CD69 activation differences between lactate conditions and the short (1 h) incubation period indicates that these phenotypic changes are not driven by differentiation‐state transitions or transcriptional reprogramming. Instead, lactate may promote CCR7 internalization and induce desensitization to this receptor.

During high‐intensity exercise, skeletal muscle releases L‐lactate into the bloodstream, creating a metabolic milieu that could modulate T‐cell bioenergetics. T‐cell subsets exhibit metabolic pathways tightly coupled to their functional roles. Upon activation and differentiation, they undergo metabolic reprogramming to meet the energetic demands of effector functions, with their metabolic profile dictating functional fate (Pearce & Pearce, 2013). Prior studies using L‐lactate concentrations (e.g., 40 mM) showed enhanced tumor infiltration, granzyme B and interferon‐γ in activated CD8^+^ T‐cells (Barbieri et al., 2023), demonstrating that lactate is a critical regulator of T‐cell function and signaling pathways in response to physiological stressors such as exercise (Barbieri et al., 2023; Cai et al., 2023). Although exciting, prior studies have used either unphysiological concentrations of L‐lactate (e.g., 40 mM) or the less physiologically relevant D‐lactate enantiomer, particularly in the context of exercise. Our findings extend this paradigm by demonstrating that even moderate, exercise‐mimicking L‐lactate concentrations (4 mM) are sufficient to enhance mitochondrial respiration in T‐cells.

In conclusion, our study demonstrated that increased lactate concentrations enhance T‐cell mitochondrial respiration, allowing them to adapt to an altered metabolic environment. A strength of the present study was that we used Plasmax™, which is specifically designed to mimic human plasma. We also used NaCl as a counterion control for the high Na^+^‐L‐lactate condition (2023), both of which were matched for osmolality within the physiological range of human plasma. However, our work has some limitations. For example, we did not investigate the roles of monocarboxylate transporters MCT1 and MCT4, which are key mediators of lactate uptake and metabolism in T‐cells. Thus, future studies are needed to examine the impact of MCT1 and MCT4 inhibitors on T‐cell bioenergetics, particularly considering that these inhibitors are potential therapeutic agents for cancer treatment (Hong et al., 2016). Additionally, our study included 12 healthy participants with moderate physical activity levels assessed through subjective ratings, which may introduce bias. It remains unclear whether these findings can be generalized to sedentary populations. Furthermore, while our *in vitro* model allowed us to isolate the direct effects of lactate on T‐cells, it does not account for the complex interplay of other exercise‐induced factors, such as exerkines, catecholamines or changes in pH. Although we did not directly measure the pH of the Plasmax media at the end of the *in vitro* experiments, Plasmax has a pH of ~7.3–7.4 after 1 h incubation at 37°C in humidified air with 5% CO_2_. Moreover, the addition of Na^+^‐L‐Lactate to Plasmax to raise the lactate concentration to 4.0 mM likely results in a slight alkalization of the media as it dissociates into the lactate anion (Miller et al., 2005). As noted in the Section 2, the pHs of the Plasmax Media with the 0.5 mM L‐lactate and 4.0 mM L‐lactate were 7.45 and 7.65, respectively, after 1 h incubation at 37°C in humidified air with 5% CO_2_. Future studies using exercise‐conditioned plasma could provide a better understanding of how lactate interacts with these factors *in vitro*. In addition, investigating *in vivo* models may offer a more comprehensive understanding of how exercise‐derived lactate influences immune function in contexts such as cancer and metabolic diseases. Despite these limitations, our findings underscore the significance of lactate in T‐cell metabolism, revealing a flexible immune‐metabolic adaptation associated with exercise.

## AUTHOR CONTRIBUTIONS

Eunhan Cho, Guillaume Spielmann and Brian A. Irving conceived and designed research. Eunhan Cho performed experiments; Eunhan Cho, Guillaume Spielmann and Brian A. Irving analyzed data. Eunhan Cho, Guillaume Spielmann and Brian A. Irving interpreted results of experiments. Eunhan Cho prepared figures. Eunhan Cho drafted the manuscript. Guillaume Spielmann and Brian A. Irving edited and revised the manuscript. All authors approved the final version of the manuscript.

## FUNDING INFORMATION

The present study was supported in part by the William Prescott Foster Endowed Professorship (BAI), the Louisiana Board of Regents Grant LEQSF(2024–25)‐ENH‐DE‐03 (BAI, GS) and unrestricted funds to GS.

## 5 ETHICS STATEMENT

All participants provided written informed consent before participation, which was approved by Louisiana State University's Institutional Review Board (IRB #24‐0411).

## Supporting information


Data S1.

## Data Availability

Data are available from the corresponding authors, Eunhan Cho and Brian Irving, upon request.
