# Supplementary material for: Effects of lactate concentration on T‐cell phenotype and mitochondrial respiration
Source: Physiol Rep. 2025 Jul 24;13(14):e70450. doi: 10.14814/phy2.70450 (PMC12288854; doi:10.14814/phy2.70450)
Supplement: Supplementary file 1 — Data S1. [file PHY2-13-e70450-s001.docx]

**Supplementary Material Table 1.** Cellular O_2_ flow (*I*_O2_, pmols∙s^-1^∙million T-cells^-1^) in T-cells that were incubated in plasma-like media with either 0.5mM or 4mM L-lactate for 1 hour, N=12, and data are presented as Mean ± 95% CI.

| Substrate State / ET Pathway State | Coupling Control  State | Lactate Concentration (Mean ± 95% CI) | | p-value |
| --- | --- | --- | --- | --- |
|  |  | 0.5mM | 4mM |  |
| CE | Routine | 1.88 (1.57, 2.20) | 2.25 (1.93, 2.57) | <0.001 |
| PalM_L_/F_L_ | LEAK | 0.56 (0.40, 0.71) | 0.71 (0.55, 0.86) | 0.015 |
| PalM_P_/F_P_ | OXPHOS | 1.84 (1.35, 2.34) | 2.26 (1.76, 2.75) | 0.005 |
| PalMPyr_P_/FN_P_ |  | 2.30 (1.84, 2.77) | 2.75 (2.29, 3.22) | 0.006 |
| PalMPyrG_P_/FN_P_ |  | 2.24 (1.82, 2.65) | 2.71 (2.29, 3.12) | 0.004 |
| PalMPyrGS_P_/FNS_P_ |  | 6.49 (5.52, 7.46) | 7.64 (6.67, 8.61) | 0.002 |
| PalMPyrGSR_P_/S_P_ |  | 5.77 (5.03, 6.52) | 6.63 (5.89, 7.38) | 0.004 |

The *I*_O2_ measured in PalM_L_/F_L_ state for one participant was excluded due to unstable readings during this phase.
